# Patients' Perceptions About the Quality of Nurses' Communication During Acute Hospitalisation: A Cross‐Sectional Survey

**DOI:** 10.1111/jan.17092

**Published:** 2025-06-03

**Authors:** Kasie Harrison, Linda Sweet, Kaara Ray B. Calma, Wendy Giddings, Brian Peralta, Tanita Botha, Amy Vaccaro, Debra Kerr

**Affiliations:** ^1^ Albury Wodonga Health Albury New South Wales Australia; ^2^ Western Health, Deakin University Partnership St Albans Victoria Australia; ^3^ Institute for Health Transformation Centre for Quality and Patient Safety Research, Deakin University Burwood Victoria Australia; ^4^ School of Nursing and Midwifery Griffith University Gold Coast Queensland Australia; ^5^ Western Health, Bacchus Marsh Hospital Bacchus Marsh Victoria Australia; ^6^ Faculty of Health Deakin University Burwood Victoria Australia

**Keywords:** acute care, adult nursing, communication, interpersonal communication, nurse–patient relationships, survey designs

## Abstract

**Aim:**

To measure patients' views of nurses' communication and interpersonal skills during acute hospitalisation.

**Design:**

This was a descriptive cross‐sectional study.

**Methods:**

From January to June 2024, a convenience sampling approach was used to recruit patients hospitalised in acute care wards across two healthcare organisations in regional Victoria, Australia. A self‐report survey, the Communication Assessment Tool for Nurses, included 15 items that measured patients' opinions about the quality of nurses' communication using a 5‐point scale. Data were analysed by descriptive and univariate statistics and logistic regression.

**Results:**

The sample included 204 participants. Higher ratings were found for respectful care: ‘Treated me with respect’ and ‘Showed care and concern’. Lower ratings largely related to shared decision‐making: ‘Encouraged me to ask questions’, ‘Informed me about my plan of care’, ‘Involved me in decisions as much as I wanted’ and ‘Showed interest in my ideas about my health’. Logistic regression revealed lower ratings for the quality of nurses' communication based on longer hospital stay for items related to greetings and shared decision‐making.

**Conclusion:**

Whilst this study found that patients perceive a high quality of respectful nursing care, the findings underscore the need for communication skills training to enhance shared decision‐making by nurses. Consideration is needed regarding how nurses are prepared to engage in shared decision‐making with patients during acute hospitalisation, particularly for longer hospital stays.

**Impact:**

This study addresses a gap in evidence regarding patients' perceptions about the quality of nurses' communication during acute hospitalisation in the Australian context. Whilst they perceive that nurses communicate in a respectful and caring manner, opportunities for shared decision‐making may not be capitalised on. Hospital managers and nurse academics should develop interventions to address essential communication skills.

**Patient or Public Contribution:**

This study did not include patient or public involvement in its design, conduct or reporting.

**Summary**

- What does this paper contribute to the wider global clinical community?
  ○ This study revealed that most patients perceive they receive respectful nursing care during acute hospitalisation.
  ○ The results indicate that there is a need to incorporate communication skills training in nursing curricula and post‐graduate training to enhance nurses' contribution to shared decision‐making with patients.

## 1 Introduction

The quality of nurses' communication is directly related to patient satisfaction and positive health outcomes (Negi et al. 2016). This is an important consideration, as generally, nurses spend more time with patients in acute care settings than any other healthcare professional group. Although some studies have examined nurses' perceptions of their communication skills using self‐report surveys (Badiyepeymaiejahromi et al. 2018; Kerr, Milnes, et al. 2020) and interviews (Allenbaugh et al. 2019; Heyn et al. 2013), evidence is scarce regarding patients' perspectives of the quality of nurses' communication.

Communication can be defined in many ways, through the way nurses act towards others (non‐verbal communication) or how they directly speak to patients, families and the multidisciplinary team (verbal communication) (Silverman et al. 2013). Non‐verbal communication usually refers to what occurs when no words are being spoken, such as body language, gestures and eye contact. In contrast, verbal communication refers to the actual content of the conversation, and the delivery style and tone of voice used. Non‐verbal communication should match verbal communication, as this is likely to build rapport between nurses and patients.

There is an expectation that nurses maintain effective communication in their praxis. In Australia, communication is specifically highlighted in both the Nursing and Midwifery Board of Australia (NMBA) (2016) Registered Nurse Standards for Practice: ‘Standard 2 Engages in therapeutic and professional relationships’, as well as the Australian Commission on Safety and Quality in Healthcare (2021): ‘Standard 2—Partnering with Consumers’. These policy documents purport that nurses should purposefully engage in effective therapeutic and professional relationships with patients, their families and the broader healthcare team. Utilisation of effective communication skills are necessary when building rapport and therapeutic relationships with patients and their families (Silverman et al. 2013). However, barriers to effective nurse–patient communication have been identified.

## 2 Background

Workplace conditions and environment, as well as patient factors, have been shown to impact the quality of nurses' communication. The most prominent barrier to nurse–patient communication relates to lack of time due to excess workload (Amoah et al. 2019; Kerr, Milnes, et al. 2020; Rachwal et al. 2018; Wune et al. 2020). Family dynamic issues have also been found to interfere with nurses' ability to communicate with patients (Kerr, Milnes, et al. 2020; Rachwal et al. 2018). Moreover, a lack of confidence and knowledge in effective communication restricts nurses' ability to communicate in challenging situations (Amoah et al. 2019; Kerr, Milnes, et al. 2020), which is compounded by factors such as cultural differences (Rachwal et al. 2018), language (Amoah et al. 2019; Kerr, Milnes, et al. 2020; Rachwal et al. 2018) and environmental issues (Amoah et al. 2019; Rachwal et al. 2018).

A key concern identified in several studies (Been‐Dahmen et al. 2015; Chan et al. 2018) suggests that patients perceive that nurses spend more time on tasks and physical care in contrast to psychosocial care. In an ethnographic study in two oncology wards in Hong Kong, Chan et al. (2018) examined patients' perceptions of nurses' communication within ward environments. Patients reported a lack of focus on psychosocial care, compared with physical care, which reduced their experience of quality nursing care. Likewise, Been‐Dahmen et al. (2015) found that nurses can overlook psychosocial challenges for patients with chronic illness in a qualitative study with 27 nurses in the Netherlands. They proposed that nurses require training to enable them to assist patients in dealing with challenging problems. Compounding this issue, it has also been identified that nurses may avoid difficult conversations, leaving patients feeling as though they do not have all the information they need (Marca‐Frances et al. 2020). This is problematic as it was identified in the same study that patients have a high level of trust in nurses and are more likely to raise concerns and doubts with nurses compared with other healthcare professionals. In a focus group study involving nurses, Chan et al. (2019) identified that nurses prioritise patients' physical needs due to excess workloads, and this impacts their ability to build rapport and communicate with patients.

Efforts to improve health outcomes and patient satisfaction for hospitalised patients include addressing the quality of patient communication by healthcare professionals. Several studies have measured the quality of nurses' communication using patient self‐report surveys (Allenbaugh et al. 2019; Belasen et al. 2021; Fite et al. 2019; Lotfi et al. 2019; Manongi et al. 2009; Sipsma et al. 2013). Using the ‘Inpatient Assessment of Health Care’ (I‐PAHC) questionnaire, Sipsma et al. (2013) measured Chinese patients' experiences of healthcare, such as communication with nurses and doctors, and pain and medication management. They found that ‘Communication with nurses’ was the factor most strongly associated with overall patients' rating of care. The authors suggested that patients' feedback about their experience is an important consideration for building a culture of patient‐centred care. The well‐known Hospital Consumer Assessment of Healthcare Providers and Systems (HCAHPS) survey instrument was used by Allenbaugh et al. (2019) and Belasen et al. (2021) to measure patients' perceptions of their hospital experience. Following the introduction of a new curriculum that focused on improving nurse communication, Allenbaugh et al. (2019) identified improvements in nurses' communication for the following items: ‘Nurses explained things in a way you could understand’ (Pre: 59%, Post 73%), ‘Nurses listened carefully to you’ (Pre: 60%, Post: 72%), ‘Nurses treated you with courtesy/respect’ (Pre: 74%, Post: 80%) and ‘Overall communication with nurses’ (Pre: 65%, Post: 75%). The study by Belasen et al. (2021) used the HCAHPS survey tool in 3382 hospitals in the United States to identify factors that shape patients' experiences within hospital settings. Significant associations were identified between overall hospital rating and care transition, nurses' communication and doctors' communication. Using the ‘Nurse Quality of Communication with Patient Questionnaire’ (NQCPQ), Lotfi et al. (2019) conducted a descriptive‐correlation study that involved patients in a burns ward in Tabriz. They found that 53% of the patient satisfaction score was correlated with the quality of nurses' communication. Lower mean scores were identified for several items relating to nurses' communication, including. ‘I feel free to ask questions’ (Mean: 3.10/6) and ‘Understands when I share my problems’ (Mean: 2.67/6). These findings are further evidence that nurses may feel challenged when interacting with patients who have psychosocial issues. In Tanzania, Manongi et al. (2009) used a parent satisfaction questionnaire in their study that evaluated the quality of nurses' communication skills with parents and families following a communication training workshop. The quality of nurses' communication improved for some survey items, including ‘Nurse didn't discuss anxieties or fears’, ‘Not easy to find someone to talk to about’, ‘Not clear who to ask for medical assistance on the ward’ and ‘Child not told what was happening when undergoing procedures.’ However, some communication survey items declined after the intervention, including ‘Nurses sometimes talked as if I wasn't there’ and ‘Not told about medication side effects’. In a further survey‐based study in Ethiopia with 192 patients, Fite et al. (2019) explored the implementation of therapeutic communication within hospital settings using a newly developed 5‐point Likert scale that included statements such as ‘Had adequate time to express my feelings and worries’ and ‘Had adequate and clear description concerning the disease and procedures’. They found that 34.9% of patients rated a high level of therapeutic communication from healthcare professionals. These findings highlight that the provision of quality communication by nurses impacts overall hospital ratings, an important consideration for healthcare leaders. In addition, there may be room for improvement in the overall quality of nurses' communication, with a focus on focused communication skills such as clarification, active listening, shared decision‐making and demonstrating respect.

Observational studies have also been used to assess the quality of nurses' communication with patients (Allenbaugh et al. 2019; Heyn et al. 2013). Heyn et al. (2013) conducted a quasi‐experimental study in Oslo, Norway, to examine the effect of the ‘Choice Assessment Tool’. The Choice Assessment Tool was designed to encourage patients to express their symptoms, voice their problems and improve clinicians' elicitation of patients' perspectives. Content analysis was undertaken using audiotaped recordings of clinician‐patient consultations (e.g., 5 physicians and 19 nurses). Analysis revealed that patients were more active participants in communication when healthcare professionals used the ‘Choice Assessment Tool’. In their pre/post‐study, Allenbaugh et al. (2019) also directly observed nurses' interactions with patients, focusing on seven communication skills, including (1) ‘The presenter and his/her title was introduced to the patient’, (2) ‘The medical information was conveyed to the patient in plain non‐medical language’, (3) ‘The provider used the phrase “What questions do you have?”’, (4) ‘The nurse was asked for updates or contributed voluntarily’, (5) ‘Used the “teach‐back” method’, (6) ‘Nurse explained the indication for new medications’ and (7) ‘Ensured complete patient understanding’. Post‐intervention, improvements in the quality of nurses' communication were observed for the following communication skills: ‘The provider used the phrase ‘What questions do you have?’ (Pre: 6%, Post: 36%, *p* < 0.0001), ‘Used the teach‐back method (Pre: 4%, Post: 22%, *p* < 0.0001) and ‘Ensured complete patient understanding’ (Pre: 27%, Post: 64%, *p* < 0.0001) (Allenbaugh et al. 2019).

There is a lack of research that has been undertaken to assess the quality of nurses' communication using non‐self‐report and observational measures. A systematic review by Kerr, Ostaszkiewicz, et al. (2020), undertaken to summarise and evaluate the effectiveness of training interventions for improving the quality of nurses' communication, included papers if they reported studies that included an intervention to improve nurses' communication skills with patients and there was an objective measure for the quality of nurses' communication either through patient‐report or observational methods. Only seven studies met the eligibility criteria, all of which used different outcome measurement tools. Also, there were no studies conducted within Australia, and most studies were conducted in palliative care settings. This current study was designed to address the gap in evidence regarding the quality of nurses' communication, particularly in acute hospital settings in Australia, and from the patient's perspective. This is an important point, as we know that nurses' communication impacts patient satisfaction with their hospital experience (Allenbaugh et al. 2019; Belasen et al. 2021). To the best of our knowledge, this is the first study to measure the quality of nurses' communication from the patient's perspective in the Australian context.

## 3 The Study

### 3.1 Aim

The aim of this study was to measure the quality of nurses' communication and interpersonal skills with patients during acute hospitalisation from the patients' perspective.

## 4 Methods

### 4.1 Design

The study was conducted using a non‐experimental, descriptive, cross‐sectional survey design. Cross‐sectional studies are used when observing a population at a single point in time, and they are often used to measure and understand health outcomes, determinants of health and describe the aspects and features of populations within healthcare fields (Wang and Cheng 2020). Non‐experimental study designs are commonly used when the results and outcomes of the study are meant to be descriptive, and the aim is to observe an activity that has already occurred. This design, therefore, suited this study as the aim was to examine how patients viewed their nurses' communication and interpersonal skills during acute hospitalisation.

### 4.2 Sample and Participants

This research project was undertaken in two separate healthcare services: one large regional health service in New South Wales (NSW) (Site A) and a smaller regional hospital in Victoria (Site B). Site A has 337 beds and services the geographical region across the border of NSW and Victoria. Data was collected at the NSW Campus in Albury, specifically two surgical wards and one medical ward with a total of 62 beds across the three wards. Site B is located in the western regional area of Victoria and has a total of 60 beds across urgent care, general medical and surgical, aged care, palliative care and maternity services. Data was collected from the medical‐surgical unit, which has a total of 24 beds.

A convenience sampling technique was used to recruit patients who were hospitalised at the two study sites between January 2024 and June 2024. The study was not designed to test a specific hypothesis, and so a formal size calculation was not conducted a priori. It was considered that a sample of at least 150 patients would provide useful information regarding the quality of nurses' communication and interpersonal skills. The term acute hospitalisation referred to a level of health care in which the patient was admitted to hospital for treatment of an episode of illness, for conditions that are the result of disease or trauma, and/or during recovery from surgery.

The Nurse Unit Managers of participating wards were asked to identify patients who met the study criteria. Patients who had been hospitalised for at least 24 h in an acute medical ward or surgical ward at the two hospital sites were eligible. Individuals who were not cognitively alert or were experiencing severe pain and clinical instability were not approached for study participation.

Patients meeting study criteria were then approached by one of the researchers (e.g., KC, BP, DK) who provided brief verbal information about the study. Patients interested in participating were then provided with a Participant Information Form, which included detailed information about what their participation would involve, the type of survey statements, data confidentiality and data management processes. After these patients had time to read the Participant Information Form (e.g., at least 15 min), they were asked if they were willing to complete the survey. Patients who completed the survey were deemed to have given implied consent. Written consent was not obtained.

### 4.3 Data Collection

A pre‐existing survey, the Communication Assessment Tool for Nurses (CAT‐N), was used to collect data for this study. The 15‐item CAT‐N is a modified version of the Communication Assessment Tool (CAT), originally designed by Makoul et al. (2007) to assess the quality of medical doctors' communication and interpersonal skills. The reliability and validity of the CAT has been evaluated (Makoul et al. 2007) and was found to have excellent reliability, with a high overall Cronbach's *α* of 0.96.

The CAT‐N was developed by the original author of the CAT (Makoul, Personal Communication, January 2022) as a tool that could be used to specifically measure the quality of nurses' communication and interpersonal skills during hospitalisation. This adapted tool, the CAT‐N, has not been formally validated. Some changes were made to the CAT in the development of the CAT‐N to increase relevance to nurses. Four statements were added: ‘Told me she/he is a nurse’, ‘Informed me about my plan of care’,’Helped me in a timely manner’ and ‘Communicated with my healthcare team.’ In addition, four statements of the original CAT were removed: ‘Understood my health concerns', ‘Gave me as much information as I wanted’, ‘Discussed next steps' and ‘Spent the right amount of time with me’. One statement was revised from ‘Staff treated me with respect’ to ‘Treated me with respect.’ Finally, an additional question related to overall care was added to the CAT‐N: ‘How would you rate the care provided to you by your nurse?’ There are no previous published studies that have reported on the use of the CAT‐N tool, and to our knowledge, has not been used in other studies.

The CAT‐N is a self‐report tool that consists of demographic information and 15 survey items: 14 statements related to the communication and interpersonal skills of nurses, and one general question (How would you rate the care provided by your nurse?). A free‐text section giving patients an opportunity to provide additional information is also included in the CAT‐N. Demographic information included age group (< 25 years, 25 to 44 years, 45 to 64 years, 65 to 84 years and ≥ 85 years), sex (male, female, not disclosed), length of hospital stay (LOS), ward type, country of birth and primary language spoken at home.

The survey was structured as a 5‐point Likert scale in which participants were asked to rank the quality of their nurses' communication for 15 items: ‘1’ for poor, ‘2’ for fair, ‘3’ for good, ‘4′ for very good and ‘5’ for excellent. Participants were asked to reflect on the nurse who cared for them in the previous shift, minimising the impact of recall bias (Wang and Cheng 2020). The survey items were originally written by Makoul et al. (2007) as simple and straightforward statements, that were accessible to patients across literacy levels. The 14 survey items related to communication and interpersonal skills of nurses included the following: (1) Told me he/she is a nurse, (2) Greeted me in a way that made me feel comfortable, (3) Treated me with respect, (4) Showed interest in my ideas about my health, (5) Paid attention to me (looked at me, listened carefully), 6) Let me talk without interruptions, (7) Informed me about my plan of care, (8) Talked in terms I could understand, (9) Checked to be sure I understood everything, (10) Encouraged me to ask questions, (11) Involved me in decisions as much as I wanted, (12) Showed care and concern, (13) Helped me in a timely manner and (14) Communicated with my healthcare team.

Participants were given a hard copy of the CAT‐N survey tool by a researcher (e.g., KC, BP, DK) who asked them to complete it within 2 h. After this time, the researcher collected the survey. In most cases, it took patients less than 10 min to complete the survey. Data was transferred from the scanned hard copy data forms to a Microsoft Excel file for analysis and storage. The hard‐copy surveys were destroyed through a secure paper shredder. Data was transferred from the Excel files into a ‘Statistical Package for the Social Sciences’ (SPSS) (IBM Corp, Released 2021) database for analysis.

### 4.4 Ethical Considerations

Ethics approval was granted by the Human Research Ethics Committees of Western Health (HREC/73089/Austin‐2021, ERM ID: 73089—21 November 2023), Albury Wodonga Health (SSA/73089/AWHEC‐2023‐402486(v1); 23 Nov 2023) and Deakin University (2023‐390; 11 Dec 2023). Participants were given a verbal and written explanation about the study, which included a reiteration of their right to choose to participate or not and an assurance of confidentiality. As previously mentioned, completion of the questionnaire was interpreted as implied consent. This study was perceived as a low‐risk study, with no collection of patient identifiers and minimal burden. Hence, written consent was not obtained.

### 4.5 Data Analysis

Data were analysed using descriptive and univariate statistics using SPSS. Participant characteristics (e.g., sex, age group, site, English language spoken at home), and LOS were considered independent variables, and survey responses (e.g., CAT‐N) were considered dependent variables.

Analysis of data showed that using a dichotomised scoring system (‘% excellent’ vs. ‘% not excellent’) was more useful as the scoring was skewed towards the ‘excellent’ end of the 5‐point Likert scale. This reflected a high ceiling effect, which means that participants' responses were clustered towards the higher end (e.g., positive responses) of the CAT‐N tool. A survey response of ‘excellent’ was coded as ‘yes’ and the remaining answers (e.g., poor to very good) was coded as ‘no’. This analysis strategy has been used in previous research by Ferranti et al. (2010) and McCarthy et al. (2013), who used the CAT survey tool to examine the communication skills of healthcare professionals. Their studies also revealed a high‐ceiling effect.

The Chi‐Square test was used to determine the statistical significance of observed differences between the two groups for categorical items (e.g., sex, age group, site and English language spoken at home). The Shapiro–Wilk test was used to determine if LOS data (continuous variable) was normally distributed. Since the normality assumption did not hold, the non‐parametric Mann Whitney *U* test was used to compare responses between the two groups. All tests were performed at a 5% level of significance. As the primary outcome was binary (‘Excellent’ vs. ‘Not Excellent’), logistic regression was used to estimate the odds for an excellent response.

### 4.6 Rigour

This study is reported in accordance with the ‘Strengthening the Reporting of Observational Studies in Epidemiology (STROBE)’ guideline. The same process was followed for each participant according to the study protocol to avoid introducing any unwanted effects or biases. To maintain external validity, there were clear and concise inclusion and exclusion criteria, and data was collected in a consistent manner by the three researchers.

## 5 Results

### 5.1 Characteristics of the Sample

The sample comprised a total of 204 participants: 151 patients (75%) at Site A and 53 patients (25%) at Site B. Regarding the ward type, there was missing data for 11 cases at Site A: Most participants were hospitalised in a surgical ward (115/140, 82%). All patients at Site B were hospitalised in a combined medical/surgical unit. One hundred and seventy‐nine participants (92.3%) reported English as their primary language spoken at home. Approximately half of the participants were female (50.5%) and aged 65 years and older (*n* = 109, 55.9%). Participants had a median LOS of 5 days (IQR: 3.0‐8.0).

### 5.2 Perceptions About the Quality of Nurses' Communication

Participant responses for the CAT‐N survey are summarised in Table 1. For the 15 CAT‐N items, the three items that received the highest rating (proportion of ‘excellent’ rating) were ‘How would you rate the care provided by your nurse?’ (73.8%), ‘Treated me with respect’ (72.1%) and ‘Showed care and concern’ (70.6%). The CAT‐N items that received the lowest ratings largely related to shared decision‐making, and included the following statements: ‘Encouraged me to ask questions’ (52%), ‘Informed me about my plan of care’ (54%), ‘Involved me in decisions as much as I wanted’ (54.0%), ‘Showed interest in my ideas about my health’ (54.4%), ‘Communicated with my healthcare team’ (54.8%) and ‘Helped me in a timely manner’ (57.5%).

**TABLE 1:** Survey responses about quality of nurses' communication.

CAT‐N item^a^	Poor	Fair	Good	Very good	Excellent
	*N* (%)	*N* (%)	*N* (%)	*N* (%)	*N* (%)
Told me he/she is a nurse	3 (1.5)	1 (0.5)	18 (8.9)	46 (22.7)	135 (66.5)
Greeted me in a way that made me feel comfortable	1 (0.5)	0 (0)	17 (8.3)	45 (22.1)	141 (69.1)
Treated me with respect	1 (0.5)	1 (0.5)	12 (5.9)	43 (21.1)	147 (72.1)
Showed interest in my ideas about my health	4 (2.0)	5 (2.5)	17 (8.3)	67 (32.8)	111 (54.4)
Paid attention to me (looked at me, listened carefully)	2 (1.0)	4 (2.0)	15 (7.4)	52 (25.5)	131 (64.2)
Let me talk without interruptions	4 (2.0)	3 (1.5)	11 (5.4)	54 (26.5)	132 (64.7)
Informed me about my plan of care	4 (2.0)	5 (2.5)	27 (13.4)	57 (28.2)	109 (54.0)
Talked in terms I could understand	3 (1.5)	2 (1.0)	17 (8.4)	43 (21.3)	137 (67.8)
Checked to be sure I understood everything	3 (1.5)	7 (3.4)	19 (9.3)	49 (24.0)	126 (61.8)
Encouraged me to ask questions	5 (2.5)	9 (4.4)	23 (11.3)	61 (29.9)	106 (52.0)
Involved me in decisions as much as I wanted	3 (1.5)	7 (3.5)	27 (13.4)	56 (27.7)	109 (54.0)
Showed care and concern	2 (1.0)	3 (1.5)	14 (6.9)	41 (20.1)	144 (70.6)
Helped me in a timely manner	1 (0.5)	4 (2.0)	24 (12.0)	56 (28.0)	115 (57.5)
Communicated with my healthcare team	6 (3.0)	3 (1.5)	21 (10.6)	60 (30.2)	109 (54.8)
How would you rate the care provided by your nurse?	3 (1.5)	1 (0.5)	12 (5.9)	37 (18.3)	149 (73.8)

^a^ Some respondents did not report data on all items. Hence, some items may not have a total number of 204 responses.

### 5.3 Comparison of Survey Responses by Participant Characteristics

Responses to the CAT‐N survey items were compared and tested for statistical significance by participant characteristics (sex, age group, English language spoken at home, site and LOS), as shown in Table 2. There were no statistically significant differences in CAT‐N survey items by sex, age group and English language spoken at home. Lower ratings (e.g., the proportion of ‘excellent’ responses) were observed at Site B (*n* = 21, 41.2%) compared with Site A (*n* = 89, 59.7%) for the CAT‐N item ‘Communicated with my healthcare team’, which reached statistical significance (*p*: 0.0327).

**TABLE 2:** Comparison of proportion of ‘excellent’ responses for quality of nurses' communication by participant characteristics.

CAT‐N item^a^	Male	Female	< 65 Year	≥ 65 Years	Site A	Site B	English	Non‐English
	*N* (%)	*N* (%)	*N* (%)	*N* (%)	*N* (%)	*N* (%)	*N* (%)	*N* (%)
Told me he/she is a nurse	67 (69.1)	63 (64.3)	61 (70.9)	69 (63.3)	103 (67.8)	32 (62.7)	121 (68.0)	9 (60.0)
Greeted me in a way that made me feel comfortable	65 (67.0)	70 (70.7)	61 (70.9)	74 (67.3)	107 (69.9)	34 (66.7)	124 (69.3)	10 (66.7)
Treated me with respect	72 (74.2)	70 (70.7)	62 (72.1)	80 (72.7)	112 (73.2)	35 (68.6)	131 (73.2)	9 (60.0)
Showed interest in my ideas about my health	52 (53.6)	55 (55.6)	49 (57.0)	58 (52.7)	87 (56.9)	24 (47.1)	98 (54.7)	8 (53.3)
Paid attention to me (looked at me, listened carefully)	65 (67.0)	61 (61.6)	59 (68.6)	67 (60.9)	102 (66.7)	29 (56.9)	115 (64.2)	10 (66.7)
Let me talk without interruptions	63 (64.9)	65 (65.7)	59 (68.6)	69 (62.7)	101 (66.0)	31 (60.8)	118 (65.9	10 (66.7)
Informed me about my plan of care	51 (53.1)	53 (54.1)	47 (55.3)	57 (52.3)	84 (55.3)	25 (50.0)	94 (52.8)	10 (71.4)
Talked in terms I could understand	65 (68.4)	67 (67.7)	57 (66.3)	75 (69.4)	107 (70.9)	30 (58.8)	122 (68.9)	9 (60.0)
Checked to be sure I understood everything	65 (67.0)	57 (57.6)	56 (65.1)	66 (60.0)	100 (65.4)	26 (51.0)	111 (62.0)	9 60.0
Encouraged me to ask questions	49 (50.5)	53 (53.5)	52 (60.5)	50 (45.5)	83 (54.2)	23 (45.1)	92 (51.4)	8 (53.3)
Involved me in decisions as much as I wanted	51 (53.1)	54 (55.1)	45 (53.6)	60 (54.5)	86 (57.0)	23 (45.1)	96 (54.2)	9 (60.0)
Showed care and concern	70 (72.2)	68 (68.7)	61 (70.9)	77 (70.0)	109 (71.2)	35 (68.6)	127 (70.8)	10 (66.7)
Helped me in a timely manner	60 (63.2)	52 (53.1)	50 (58.1)	62 (57.9)	83 (55.7)	32 (62.7)	100 (56.8)	12 (80.0)
Communicated with my healthcare team	59 (61.5)	47 (49.0)	45 (52.3)	61 (57.5)	89 (59.7)^#^	21 (41.2)	98 (56.0)	8 (53.3)
How would you rate the care provided by your nurse?	72 (74.2)	72 (74.2)	62 (73.8)	82 (74.5)	114 (75.5)	35 (68.6)	132 (73.7)	11 (73.3)

^a^ Some respondents did not report data on all items. Hence, some items may not have a total number of 204 responses.

^#^ Statistically significant difference, *p* = 0.0327.

The proportion of ‘excellent’ responses to CAT‐N survey items was compared by median LOS, as shown in 3. As previously described, data was not normally distributed, hence, the Mann–Whitney *U*‐Test was used to compare results between the two groups. Regarding the CAT‐N survey item ‘Involved me in decisions as much as I wanted’, participants who reported an ‘excellent’ response had a shorter LOS (*n* = 98, median 4.0 days) compared with participants who did not report an ‘excellent’ response (*n* = 87, median 5.0 days, *p*: 0.0434, as shown in Table 3).

**TABLE 3:** Comparison in median length of stay for ‘excellent’ responses for quality of nurses communication.

CAT‐N item	Excellent response	*N*	Median (IQR)	*p*
Told me he/she is a nurse	Yes No	124 62	5.0 (3,7) 5.0 (3,9)	0.3161
Greeted me in a way that made me feel comfortable	Yes No	126 61	4.0 (3,7) 6.0 (3,10)	0.1034
Treated me with respect	Yes No	137 50	5.0 (3,7) 5.5 (3,9.5)	0.2087
Showed interest in my ideas about my health	Yes No	104 83	5.0 (4,8) 4.0 (3,8)	0.1958
Paid attention to me (looked at me, listened carefully)	Yes No	120 67	5.0 (3,7.25) 5.0 (3,8)	0.7445
Let me talk without interruptions	Yes No	123 64	5.0 (3,7) 5.0 (3,8.3)	0.2077
Informed me about my plan of care	Yes No	99 86	4.0 (3,7) 5.0 (3,8.8)	0.0605
Talked in terms I could understand	Yes No	124 62	5.0 (3,8) 5.0 (3,8)	0.7339
Checked to be sure I understood everything	Yes No	116 71	5.0 (3,7) 5.0 (3,9.5)	0.2983
Encouraged me to ask questions	Yes No	97 90	5.0 (3,7) 5.0 (3,9)	0.3517
Involved me in decisions as much as I wanted	Yes No	98 87	4.0 (3,7) 5.0 (3,9)	0.0434*
Showed care and concern	Yes No	133 54	5.0 (3,7) 5.0 (3,10)	0.4366
Helped me in a timely manner	Yes No	107 79	4.0 (3,7) 5.0 (3,8)	0.1364
Communicated with my healthcare team	Yes No	101 83	4.0 (3,7) 5.0 (3,9.5)	0.0897
How would you rate the care provided by your nurse?	Yes No	137 48	5.0 (3,7) 5.5 (3, 10.8)	0.1858

* Statistically significant difference, *p* = 0.0434.

Logistic regression was used to test for independent associations, controlling for gender, age group, site, English language at home and LOS. As LOS had large outliers, the variable was log transformed to decrease the effect on the model. Length of stay affected responses to eight survey items, as shown in Table 4: ‘Told me he/she is a nurse’ (*p*: 0.0376), ‘Greeted me in a way that made me feel comfortable’ (*p*: 0.0218), ‘Treated me with respect’ (*p*: 0.0239), ‘Informed me about my plan of care’ (*p*: 0.0262), ‘Checked to be sure I understood everything’ (*p*: 0.0240), ‘Involved me in decisions as much as I wanted’ (*p*: 0.005) and ‘Communicated with my healthcare team’ (*p*: 0.004). Participants with a longer LOS also provided lower ratings for the question ‘How would you rate the care provided by your nurse?’ (*p*: 0.025).

**TABLE 4:** Factors associated with an ‘excellent’ response.

CAT‐N item		Odds ratio^a^	95% CI	*p*
Told me he/she is a nurse	LOS	0.643	0.418, 0.969	0.0376
Greeted me in a way that made me feel comfortable	LOS	0.611	0.394, 0.922	0.0218
Treated me with respect	LOS	0.606	0.385, 0.928	0.0239
Informed me about my plan of care	LOS	0.623	0.403, 0.932	0.0262
	Site B	0.467	0.220, 0.976	0.0445
Checked to be sure I understood everything	LOS	0.619	0.399, 0.928	0.024
Encouraged me to ask questions	Age ≥ 65	0.522	0.284, 0.948	0.0341
Involved me in decisions as much as I wanted	LOS	0.533	0.337, 0.813	0.005
Communicated with my healthcare team	LOS	0.520	0.324, 0.801	0.004
How would you rate the care provided by your nurse?	LOS	0.603	0.381, 0.928	0.025

Abbreviation: LOS, length of stay.

^a^ The reference group for the odds ratio is an ‘excellent’ response.

Participants at Site B were less likely to report an excellent response to the statement ‘Checked to be sure I understood everything’ compared with participants at Site A (Odds Ratio 0.467, 95% CI: 0.220, 0.976, *p*: 0.0445). Regarding the statement ‘Encouraged me to ask questions’, older age (≥ 65 years) was found to be associated with a lower likelihood of an excellent response (Odds Ratio: 0.522, 95% CI: 0.284, 0.948, *p*: 0.0341).

## 6 Discussion

This study addresses a gap in evidence regarding patients' perceptions of the quality of nurses' communication in the Australian context. Overall, patients in this study reported that nurses' communication is of a high standard: Nurses demonstrate respect and concern for patients in their care. Findings, however, suggest that there is scope for nurses to foster shared decision‐making by involving patients in decisions about their care. There is limited research that has been undertaken to measure the quality of nurses' communication using observational or patient‐report measures, as identified by a recent systematic review by Kerr, Ostaszkiewicz, et al. (2020). This study addresses this gap.

Analysis revealed that patients perceive that nurses provide respectful care, with highest ratings for the items ‘Treated me with respect’ (72.1%) and ‘Showed care and concern’ (70.6%). This suggests that nurses are meeting recommendations outlined by Silverman et al. (2013) in the ‘Calgary‐Cambridge Guides Communication Process Skills’, where it is proposed that healthcare professionals should demonstrate respect and interest in their patients' health. Our findings correlate with the interventional study conducted in Philadelphia, United States, by Allenbaugh et al. (2019), with similar ratings for the provision of respect and courtesy in the pre‐ (74%) and post‐ (80%) phases, measured through direct observation. In contrast to these findings, Kannan et al. (2020) found low ratings for respectful nursing care in their study based in South India, in which the ‘Patient Satisfaction with Nursing Care Quality Questionnaire’ was used in a cross‐sectional descriptive study. Patient ratings of patient satisfaction were low for several items, including the following: ‘Care and concern shown by nurse’ (40%), ‘Helpful attitude of nurse towards patient’ (49%) and ‘Attention by nurse towards patients’ condition’ (34%). In a nurse self‐report study, Badiyepeymaiejahromi et al. (2018) identified that 94% of nurses reported poor provision of respectful nursing care. Differences observed in our current study with these two studies (Kannan et al. 2020; Badiyepeymaiejahromi et al. 2018) may reflect factors related to the diverse geographical regions and cultural differences. Also, data was collected for these studies using different techniques (observation and nurse self‐report survey) which may affect the reliability of study findings. However, our research demonstrates consistency with three previous studies (McCarthy et al. 2013; Makoul et al. 2007; Ferranti et al. 2010), all which utilised various forms of the CAT survey tool. For all four studies, including this reported study, the item ‘Treated me with Respect’ was one of the top three rated survey items. Conversely, the items ‘Encouraged me to ask questions' and ‘Involved me in decisions as much as I wanted’ were within the bottom three rated survey items.

Evidence suggests that nurses and other healthcare professionals are less efficient in involving patients in decisions about their care: Shared decision‐making. In a descriptive‐correlational study by Lotfi et al. (2019), it was reported that patients were dissatisfied with nursing care related to the following items: ‘Fails to consider my opinions and preferences regarding plans for my care’ and ‘Neglects to be sure I understand the importance of my treatments’. Ferranti et al. (2010) and McCarthy et al. (2013) also found low ratings for shared decision‐making items, including ‘Showed interest in my ideas about my health’ (58%, 59%), ‘Encouraged me to ask questions’ (53%, 50%) and ‘Involved me in decisions as much as I wanted’ (53%, 55%), respectively. However, these studies did not specifically examine nurses' communication. For example, the study by McCarthy et al. (2013) was undertaken to explore the quality of the emergency department teams' communication and did not differentiate the quality of communication between individual healthcare professional groups, such as nurses and medical doctors. Despite these limitations, previous research suggests some consistency in the capacity of healthcare professionals to engage with patients in shared decision‐making.

There is an emphasis on shared decision‐making within legal (e.g., consenting procedures), policy and theoretical literature. It is purported in two Australian professional guidelines, the ‘National Safety and Quality Health Service Standards—Partnering with Consumers’ (Australian Commission on Safety and Quality in Healthcare 2021) and the ‘Registered Nurse Standards for Practice—Engages in therapeutic and professional relationships’ (NMBA 2016), that healthcare professionals, including nurses, should work alongside and empower patients to be as involved as possible in their own care. A legal ruling in the United Kingdom resulting from the Montgomery v. Lanarkshire Health Board (Ward et al. 2020) established the importance of shared decision‐making in the consent process. In the view of the Supreme Court judges, and in light of contemporary standards of shared decision‐making, a mother with gestational diabetes should have been advised about the risks of shoulder dystocia to inform her decision about preferred birthing options. Researchers have included shared decision‐making in person‐centred practice frameworks (McCormack and McCance 2016) and communication interventions (e.g., team‐talk) (Elwyn et al. 2017) as an important communication skill that enables negotiation and working together to reach a consensual decision between the healthcare professional and the patient. Whilst it has been found that nurses and patients both acknowledge the importance of shared decision‐making, challenges exist in applying it in practice (Mills et al. 2025).

Communication skills to improve shared decision‐making between nurses and patients can improve with training. Chan et al. (2019) identified that communication skills training could provide new nurses with the required skills to provide a more holistic style of care, with adequate consideration of patients' psychosocial needs as well as physical needs. In their quasi‐experimental study in Victoria, Kerr, Ostaszkiewicz, et al. (2020) identified improvement in self‐assessed capacity to involve patients in shared decision‐making following engagement in a communication skills training program. The strength of those study findings may be limited as self‐report measures were collected from nurses, and previous studies have shown that observational methods of data collection may be more reliable when measuring behaviour (Ampt et al. 2007). Confirming this limitation, Légaré et al. (2018) were unable to confirm that targeted interventions increase the use of shared decision‐making by healthcare professionals in a Cochrane systematic review that only included observer‐based or patient‐reported measures. Hence, it could be suggested that strategies are needed to increase nurses' skills in involving patients in decisions about their care.

It was identified in this study that older patients (> 65 years of age) are more likely to report that nurses encouraged them to ask questions compared with younger patients (< 65 years of age). This contrasts with the findings in a study by Podder et al. (2024), in which no statistical association between age and patient satisfaction with nurses' communication was identified in their cross‐sectional study. The findings of this study also indicated that patients with longer hospitals stays were less likely to feel that nurses involved them in decision‐making to the extent they desired. Podder et al. (2024) also found that shorter hospital duration is associated with higher patient satisfaction with nursing care. The scale incorporated in their study included three sub‐domains of patient satisfaction: Information, Education and Communication. The association between patient satisfaction and LOS was calculated using the total satisfaction score. Hence, it is not clear whether communication was a significant independent factor in their study. The reasons for our observation of lower ratings for the item ‘Informed me about my plan of care’ at Site B requires further investigation.

### 6.1 Strengths and Limitations of the Work

The findings of this study contribute to new knowledge regarding the quality of nurses' communication, particularly in the Australian and acute care contexts, where there is a dearth of research in this field (Kerr, Ostaszkiewicz, et al. 2020). This study involved data collection by one researcher at each site following a strict protocol. An advantage of the CAT‐N survey tool is that it is brief and took participants less than 5 min on average to complete. Hence, patients experienced low burden through study participation. This study partly addresses the gap in evidence about patients' perceptions of the quality of nurses' communication during acute hospitalisation.

This study has several limitations. The response rate was quite low and may reflect response bias. This study was conducted at a rural and regional hospital in the state of Victoria, Australia. Therefore, the findings may not reflect the experiences of patients hospitalised in other states in Australia and overseas and larger metropolitan healthcare settings. This is an important consideration as Garcia‐Lacalle and Martin (2010) have found that patients may experience higher satisfaction with hospital care in rural hospitals compared to metropolitan hospitals.

A larger sample was obtained from the rural health service, which reflected the larger organisation. This may have affected observed differences between the two sites. There was a poor response to the open‐ended survey question regarding additional feedback. Participants who did leave comments reported on the quality of nursing care rather than specifically commenting on the quality of nurses' communication. Hence, open‐ended responses were not analysed for this study.

Previous studies have identified that the CAT‐T (McCarthy et al. 2013) and the CAT (Ferranti et al. 2010) can result in responses that reflect a high ceiling effect, also observed in this study. However, analysing the ‘excellent’ vs. ‘not excellent’ responses, as applied by McCarthy et al. (2013) and Ferranti et al. (2010), allowed for comparing findings with those studies. Lastly, the sample was largely older, hospitalised within a surgical ward and spoke English as their primary language at home. Hence, findings may not reflect the experiences of younger people, those hospitalised in other settings outside surgical wards (e.g., critical care areas, emergency departments and speciality areas), and people who prioritise other languages at home.

### 6.2 Recommendations for Further Research

There has been a lack of research exploring exactly what communication skills are needed by nurses, particularly in the acute hospital settings outside oncology and palliative care. Larger, multi‐centre studies are needed to confirm the findings of this study, particularly in metropolitan hospital and general medical/surgical ward settings. Further adequately powered studies are needed to analyse the effect of individual nursing‐related (e.g., educational preparation, age, duration of experience and attitudes) and patient‐related (e.g., length of hospital stay, age, ward type) factors on the quality of nurses' communication and interpersonal skills. The survey tool, the CAT‐N, has not been psychometrically tested, which could also be a focus for future studies. Examination of the literature has revealed that there is a lack of consistency for objective measurement of communication skills for nurses (Kerr, Ostaszkiewicz, et al. 2020), with a range of non‐validated and reliable tools previously utilised. For this study, we sought a tool that was suitable for participants who may be unwell, with key features including plain language and brevity. It is feasible that responses may have been affected by the patients' condition during hospitalisation, leading to recall bias. Longitudinal studies may address this gap and provide valuable information regarding the reliability for measurement of nurses' communication across the hospitalisation period. Although likely to be resource‐intensive, observational studies using audio or video recordings are needed to accurately measure the application of effective communication skills by nurses in acute hospital settings.

### 6.3 Implications for Policy and Practice

Whilst this study found that patients perceive a high quality of respectful nursing care, the findings underscore the need for communication skills training in preparatory courses and professional development initiatives to enhance shared decision‐making by nurses with their patients. Consideration is needed regarding how nurses are prepared to engage in shared decision‐making with patients during acute hospitalisation, particularly for longer hospital stays. It is feasible that some nursing programs include content related to communication skills training. However, it is the authors' experience that most programs have minimal focused content, particularly with the inclusion of experiential learning, and it is rare for the inclusion of unique units/modules in communication skills training. Hospital managers, nurse academics and consumers should work together using co‐design methods to develop effective and feasible interventions to improve essential communication skills for nurses. A starting point could be an identification of the essential communication skills needed by nurses.

## 7 Conclusions

Providing quality communication for patients within acute hospital settings plays an integral role in providing quality person‐centred care. The findings of this study address a gap in evidence for quality of nurses' communication in acute care and Australian contexts. This study found that, overall, nurses are providing communication that is respectful and demonstrative of caring practice. However, nurses may be less confident in communicating with patients when making decisions about or planning care. Larger observational studies, undertaken in metropolitan Australian settings, are needed to confirm the findings identified in this current study. Interventions are needed to improve nurses' confidence in shared decision‐making. The findings of this study support the call for more attention to be given to behavioural skills, such as communication skills, in nursing curriculum and post‐graduate training initiatives.

## Author Contributions

All authors have agreed on the final version and meet at least one of the following criteria (recommended by the ICMJE*): (1) substantial contributions to conception and design, acquisition of data, or analysis and interpretation of data; (2) drafting the article or revising it critically for important intellectual content. K.H., L.S., K.R.B.C., W.G., A.V., D.K. made substantial contributions to conception and design, or acquisition of data, or analysis and interpretation of data. K.H., L.S., K.R.B.C., W.G., B.P., T.B., A.V., D.K. involved in drafting the manuscript or revising it critically for important intellectual content. K.H., L.S., K.R.B.C., W.G., B.P., T.B., A.V., D.K. given final approval of the version to be published. Each author should have participated sufficiently in the work to take public responsibility for appropriate portions of the content. K.H., L.S., K.R.B.C., W.G., B.P., T.B., A.V., D.K. agreed to be accountable for all aspects of the work in ensuring that questions related to the accuracy or integrity of any part of the work are appropriately investigated and resolved.

## Disclosure

Statistical analysis: There is a statistician on the author team: T.B.

## Conflicts of Interest

The authors declare no conflicts of interest.

## Data Availability

The data that support the findings of this study are available from the corresponding author upon reasonable request.
